# Current Nutritional Therapies in Inflammatory Bowel Disease: Improving Clinical Remission Rates and Sustainability of Long-Term Dietary Therapies

**DOI:** 10.3390/nu15030668

**Published:** 2023-01-28

**Authors:** Elizabeth A. Reznikov, David L. Suskind

**Affiliations:** Division of Gastroenterology, Department of Pediatrics, Seattle Children’s Hospital, University of Washington, Seattle, WA 98105, USA

**Keywords:** inflammatory bowel disease, Crohn’s disease, ulcerative colitis, exclusive enteral nutrition, Crohn’s disease exclusion diet, specific carbohydrate diet, FODMAP, Mediterranean diet

## Abstract

Inflammatory Bowel Disease (IBD) includes a spectrum of chronic immune-mediated intestinal diseases thought to be related to the complex interaction between the host immune system and the intestinal microbiome. Research supports the use of nutritional therapy in IBD; however, it is not routinely used in clinical practice. This literature review seeks to advance the understanding of diet and its effect in IBD with a focus on both Crohn’s Disease (CD) and Ulcerative Colitis (UC). The contribution of diet to the development and treatment of IBD cannot be overstated. In both pediatric as well as adult IBD, nutritional interventions have been shown to improve clinical symptoms as well as inflammatory burden. The impact of dietary intervention is best exemplified through the use of Exclusive Enteral Nutrition (EEN) in CD. EEN and clinical research on exclusionary whole food diets—Crohn’s Disease Exclusion Diet (CDED), Specific Carbohydrate Diet (SCD), low fermentable oligosaccharides, disaccharides, monosaccharides and polyols (FODMAP) diet, and Mediterranean Diet—are discussed within this review. Current clinical literature supports the elimination of detrimental components and the incorporation of low processed whole foods in the diet. Additional prospective and longitudinal dietary studies on sustainable and long-term dietary options, along with a deeper understanding of the mechanism, are needed to further advance the role of nutritional interventions in IBD.

## 1. Introduction

Inflammatory Bowel Disease (IBD) includes a spectrum of chronic immune-mediated intestinal diseases. While the etiopathogenesis of IBD is not completely understood, it is thought to be interrelated to the complex interaction between the host immune system and the intestinal microbiome. Given the increased prevalence of IBD in countries that adopt a Western lifestyle, prevailing theories propose a significant contribution of the Western diet to the development of IBD [1]. To further this point, studies have shown that dietary exposures impact disease course in IBD [1,2]. This paper reviews the current dietary approaches utilized in Crohn’s Disease (CD) and Ulcerative Colitis (UC) as treatments for both clinical symptoms as well as the inflammatory process itself (Table 1). We searched the PUBMED database and clinical trials registries to review relevant studies. Published trials that compared dietary interventions for the management of inflammatory bowel disease with other active interventions or standard therapy, placebo, or no therapy were included.

The etiopathogenesis of IBD involves impaired intestinal barrier function and alteration of the composition and function of the intestinal microbiome with associated upregulation of the intestinal immune system [3]. These findings are also linked to diet in both animal and human studies. Dysbiosis, or altered intestinal microbiome, is a commonly found in IBD patients [4,5]. Patients with CD are noted to have increased abundances of *Enterobacteriaceae, Pasteurellacaea, Veillonellaceae,* and *Fusobacteriaceae*, and decreased abundances of *Erysipelotrichales, Bacteroidales*, and *Clostridiales* [6]. In UC, the dysbiosis is associated with low phylotype diversity and the depletion of commensal bacteria with an overabundance of *Enterobacteriaceae* and *Enterococcus* and under-representation of *Ruminococcus* and *Bacteroides* [7,8,9]. Diet also has a significant influence the intestinal mucosal barrier [10]. In an IBD mice model, animals fed a high fat and sugar diet had dysbiosis with an overabundance of *E. coli* with associated breakdown of the mucous layer and increased permeability [11]. In addition, specific food additives common in a Western diet have been shown to influence intestinal inflammation. Exposure to low concentrations of polysorbate 80, a common food emulsifier, increased translocation of adherent-invasive *E. coli* (AIEC) across intestinal M cells and Peyer’s patches with a resultant inflammatory response [12]. Dietary polysaccharide maltodextrin, a common food additive, has been associated with enhanced AIEC biofilm formation [13]. Similar effects of diet on the intestinal microbiome and mucosal integrity have been seen in human studies. In an interventional human study, significant changes in microbiome composition occurred within a day of changing diet, underlying the malleable nature of the intestinal microbiome and its link to diet [14]. Randomized controlled studies in humans also suggest that food additives such as emulsifiers and thickeners likely contributed to intestinal inflammation [15,16]. Taken together, there is ample evidence for a substantial impact of diet on intestinal microbiomes and epithelial barrier function, suggesting a potential mechanism of action for dietary intervention in IBD.

The Selecting Therapeutic Targets in Inflammatory Bowel Disease (STRIDE) initiative of the International Organization for the Study of Inflammatory Bowel Disease proposed treatment targets in 2021 including both adult and pediatric IBD [17]. STRIDE-II focused on both short-term and long-term/maintenance targets. It is important to understand these target measures when discussing interventions including dietary therapies in IBD. Symptom resolution is the main short-term treatment target with normalization of inflammatory labs as intermediate targets. The main long-term targets include restoration of growth and mucosal healing. In CD, clinical symptoms do not always correlate with the degree of mucosal inflammation seen on endoscopy. Treatment is guided by a composite strategy of symptom monitoring through clinical activity indices such as the Pediatric Crohn’s Disease Activity Index (PCDAI)/Crohn’s Disease Activity Index (CDAI) scores and Harvey–Bradshaw Index (HBI), in conjunction with objective measures of inflammation including biochemical markers (fecal calprotectin and C-reactive protein (CRP)), and restaging colonoscopy. Unlike CD, clinical symptoms (stool frequency, rectal bleeding) have better correlation with endoscopic inflammation in UC. In UC, the Pediatric Ulcerative Colitis Activity Index (PUCAI)/Mayo score is used as a clinical score, and the Mayo Endoscopic Score (MES) is used for mucosal healing. Throughout this review of the current clinical literature, analysis of the dietary interventions will use these metrics.

## 2. Exclusive Enteral Nutrition

A first-line treatment for the induction of remission for mild-to-moderate pediatric CD is exclusive enteral nutrition (EEN), which is the most established, evidence-based dietary therapy used in IBD [18]. EEN therapy is defined as the use of a complete nutritional formula as sole dietary intake over 6–10 weeks. This has been shown to achieve clinical and biochemical remission in approximately 80% of pediatric CD patients with significantly improved endoscopic mucosal healing as compared to corticosteroids [19,20,21,22,23]. Borelli et al. and Pigneur et al. reported mucosal healing rates of 73% and 89%, respectively. Adult CD studies on EEN induction of remission have shown overall less efficacy. While this may be related to differences in disease expression, it is more likely secondary to compliance. While most of adult EEN studies allowed for oral intake of formula, the adult studies which required nasogastric tube placement for EEN showed similar efficacy to pediatric studies [24,25]. Another area of growing interest with EEN is its role in pre-operative optimization in CD. While the current research is retrospective, its implications on outcome may be significant [26]. While EEN therapy has been shown to induce remission, long-term therapy with EEN has limited utility given the concerns of “formula fatigue”. 

There is no current data to support the routine use of EEN for the induction of remission in UC, although EEN may provide benefits to UC patients by improving symptoms and nutritional status [27,28]. Further assessment of EEN’s role in UC requires additional research [29].

The composition of formulas used as EEN varies greatly [30]. To date, no formula has shown any benefits over any other formula in inducing clinical remission in CD [31,32]. A compositional analysis on 61 formulas successfully used as EEN to induce remission in active CD, as compared to dietary intake of children with longstanding CD, revealed that formulas used as EEN generally contained lower sugar, total fat, saturated fat, and fiber content, and a higher percentage of protein [30]. Food additives in EEN did not affect remission rates. This may imply that content as well as dietary monotony play a role in efficacy of EEN. 

While the mechanism by which EEN induces remission is still under debate. EEN’s impact on the intestinal microbiome is well documented, although at times divergent to current scientific dictums. For example, EEN treatment decreases intestinal fecal bacterial diversity in contrast with the common belief that diverse microbiomes are important to health [33,34]. With that being noted, EEN-induced remission leads to higher rates of mucosal healing as compared to steroids. Pigneur et al. showed that this was associated with a different intestinal microbiota composition compared to corticosteroids [22]. Similarly, EEN induces the paradoxical enrichment in Firmicutes bacterial species connected with Crohn’s disease, including *Ruminococcus gnavus*, *Ruminococcus torques*, and several *Clostridium* species [22,35,36]. EEN is also associated with a fecal decrease in the Bifidobacterium species *Faecalibacterium prausnitzii,* which is thought to play an important role in intestinal homeostasis [37,38]. Although these shifts in the intestinal microbiome are contrary to scientific expectations, these shifts are still likely important with regard to the mechanisms by which EEN works. This is highlighted in an IL-10 knockout mouse model where carboxymethylcellulose and polysorbate-80, two common food emulsifiers, induced intestinal inflammation in animals with intact microbiome but not in germ-free mice [39]. Other potential mechanisms by which EEN induces mucosal healing include the direct effect on the inflammatory cascade pathway and hormones such as serum insulin-like growth factor 1 (IGF-1), serum transforming growth factor-beta (TGF-β1), and decreased serum vascular endothelial growth factor (VEGF) [27,40,41]. Wedrychowicz et al. was also able to show EEN stimulated TGF-β1 in patients with CD, but not in UC, and patients with CD achieved faster disease remission [27]. Taken together, these serum changes support an anti-inflammatory systemic effect, and are postulated to explain the mucosal healing in patients on EEN.

## 3. Partial Enteral Nutrition (PEN) with and without an Exclusion Diet 

Partial enteral nutrition (PEN) is defined by the dietary intake of between 50% and 90% of calories from formula and the rest by whole foods. Johnson et al. studied the effect of PEN as compared to EEN to understand the impact of a regular diet in active pediatric CD. Fifty children with active CD were randomized to receive 50% PEN or EEN. While both groups had improved symptoms and nutritional benefit, those on PEN had a significantly lower remission rates (15 vs. 42%) [42]. In addition, the EEN group as compared to the PEN group showed significant improvement in laboratory measures including increased albumin and decreased erythrocyte sedimentation rate (ESR). This highlights the impact of a regular diet on the inflammatory burden in CD. 

To further understand diet’s role in CD, Crohn’s Disease Exclusion Diet (CDED) was paired with PEN. CDED is a whole food diet intended to limit exposure to foods that are believed to negatively impact the intestinal microbiome, alter intestinal barrier function, or induce colonic inflammation. There is a general emphasis on high-quality lean protein, resistant starch, and moderate fiber, while avoiding high fat, dairy, high sugar, artificial additives, and emulsifiers (Table 2). CDED is combined with varying amounts of PEN over time including an induction and maintenance phase. Clinical trials have shown remission rates in children and adults similar to that of EEN [43,44,45]. Levine et al. conducted a 12-week prospective pediatric trial in active CD with patients randomized to PEN with CDED vs. EEN [43]. Both PEN with CDED and EEN resulted in high rates of remission and decreased inflammation at six weeks with no significant difference between the groups. At week six, 30 (75%) out of 40 children given CDED plus PEN were in steroid-free remission versus 20 (59%) out of 34 children given EEN (*p* = 0.38). CDED plus PEN had superior tolerance to EEN as defined by lower withdrawal from the study (97.5% vs. 73.6%).

Sigall Boneh et al. demonstrated that CDED may be used as a rescue treatment for patients with CD who have failed biologic therapy [46]. A small study was conducted in 11 adults and 10 children, and clinical responses were obtained in 19 out of 21 patients with remission in 13 measured by the HBI. The mean decrease in HBI was 9.4 ± 4.2 to 2.6 ± 3.8 (*p* < 0.001). Patients had a mean decrease in CRP (2.8 ± 3.4 to 0.7 ± 0.5) at the 12-week follow-up. Recently, Yanai et al. completed an adult pilot study of CDED with/without PEN and showed it to be effective in inducing and maintaining remission in adults with mild-to-moderate biologic naive Crohn’s disease. At week six, sixty-eight percent of patients in the CDED plus partial enteral nutrition group and fifty-seven percent of patients in the CDED group had achieved clinical remission. Among patients in remission at week 6, eighty percent had sustained remission at week 24. Thirty-five percent of patients were in endoscopic remission at week 24 (eight patients in the CDED plus partial enteral nutrition group and six in the CDED alone group) [45].

CDED with PEN is associated with significant alterations in both the intestinal microbiome as well as metabolome. In a recent study by Verburgt et al., CDED with PEN or EEN-induced remission was associated with decreased abundance of Proteobacteria and increased Firmicutes. A mixture of two metabotypes denoted as M1 and M2 were seen in CD patients, whereas all healthy controls had metabotype M1. M1 was categorized by high Bacteroidetes and Firmicutes, low Proteobacteria, and higher small-chain fatty acid (SCFA) synthesis, and M2 was associated with high Proteobacteria and SCFA degradation. For CD patients, M1 contribution increased from 48% at baseline to 74% at 12 weeks [47]. CDED with PEN as well as EEN-induced remission are also associated with significant changes in metabolites linked to active CD such as kynurenine and ceramides [48]. The findings of PEN with CDED support the concept of dietary exclusion to maintain remission and offer an alternative and more sustainable option to EEN that would make maintenance of dietary intervention in IBD possible. 

## 4. Whole Foods and Exclusion Diets 

With continued evidence of the impact of diet in IBD, the International Organization for the Study of Inflammatory Bowel Disease (IOSIBD) reviewed current evidence of potentially harmful and beneficial dietary components in CD and UC [49]. For CD, regular intake of fruits and vegetables was recommended in the absence of stricturing disease. In UC, increased consumption of natural sources of omega-3 fatty acids (not from supplements) was potentially beneficial. For both CD and UC, the recommendation was to reduce saturated fats, trans fats, dairy fats, additives (such as polysorbate 80 and carboxymethylcellulose), processed foods rich in maltodextrins, and artificial sweeteners containing sucralose or saccharine. The IOSIBD also noted that patients with persistent symptoms, despite resolution of inflammation, may have symptoms that benefit from a low FODMAP or lactose-free diet. The IOSIBD noted that research was lacking in terms of making recommendations with regard to intake of gluten, poultry, alcohol, or refined sugars; further randomized controlled trials were felt necessary. 

## 5. Hereinafter, We Will Discuss Ongoing Clinical Research on Exclusionary Whole Food Diets: SCD, FODMAP, and Mediterranean Diet

### 5.1. Specific Carbohydrate Diet

In the 1930s, pediatrician Dr. Sydney Haas developed the Specific Carbohydrate Diet (SCD) as a treatment for patients with celiac disease. This diet excludes all grains, sugars (except for honey), processed foods, and dairy (aside from fully fermented yogurt and some hard cheeses). SCD was popularized in the 1990s after Elaine Gottschall’s daughter was successfully treated for UC. In addition, there is strong support for SCD in the IBD patient population. In an anonymous survey of 417 pediatric and adult patients with IBD on the SCD, 36% reported clinical remission by one to three months, with an additional 34% at greater than three months. Of those who reached remission, 47% reported improvement in abnormal laboratory values [50].

Suskind et al. completed the first chart review of the SCD in active pediatric CD at Seattle Children’s Hospital from 2005 to 2012. Patients had resolution of symptoms in conjunction with normalization of albumin, anemia, fecal calprotectin, and CRP within 12 weeks [51]. Subsequently, Cohen et al. showed small bowel mucosal healing on capsule endoscopy by 12 weeks of SCD in nine pediatric CD patients [52]. Another retrospective chart review from Seattle Children’s Hospital from 2012 to 2014 examined the outcomes of twenty CD and six UC patients on SCD [53]. Patients with CD had improved PCDAI scores, improved ESR, CRP, and fecal calprotectin levels on SCD. Patients with UC had improved PUCAI scores, and laboratory values improved or remained normal on SCD. Despite clinical and laboratory improvement seen on the SCD, nine patients experienced weight loss on the diet and many patients found it difficult to maintain, necessitating liberalization of the diet to include rice, oatmeal, potatoes, and cocoa powder. 

A 12-week prospective study by Suskind et al. in active pediatric CD and UC confirmed the clinical and biochemical improvement found with the SCD as well as noted significant changes in microbial composition following SCD. Both the mean PCDAI and PUCAI normalized at 12 weeks from 28.1 ± 8.8 to 4.6 ± 10.3 and 28.3 ± 23.1 to 6.7 ± 11.6, respectively. Dietary therapy was not effective for two patients with two individuals not able to maintain the diet. Mean CRP also normalized at 12 weeks from 24.1 ± 22.3 to 7.1 ± 0.4 mg/L in Seattle patient cohort (normal < 8.0 mg/L) and 20.7 ± 10.9 to 4.8 ± 4.5 mg/L in Atlanta patient cohort (normal < 4.9 mg/L) [54]. 

To better understand the impact of the degree of dietary exclusion on clinical outcomes, Suskind et al. performed a small prospective randomized study of the SCD, modified SCD, and a healthy whole foods diet in active CD. With each diet, symptoms and inflammatory burden improved with the more exclusionary diets being associated with better resolution of inflammation [55]. Following this study, the PRODUCE study, a large multicenter study examining diet effect in active pediatric IBD, compared the SCD versus modified SCD [56]. The PRODUCE study utilized an *n-of-1* methodology over a 32-week period. While showing that the SCD and MSCD had benefits over standard diet for many patients with regard to symptoms and calprotectin, the study did not show a significant difference between the two diets. The DiNE-CD, an adult study in active CD, showed symptomatic improvement with both SCD and Mediterranean diets (MD) in active CD [57]. At six weeks, the percentage of patients achieving symptomatic remission was no different between SCD and MD (SCD, 46.5%; MD, 43.5%; *p* = 0.77). In addition, no difference was seen in fecal calprotectin response between patients on the SCD (34.8%) and MD (30.8%) (*p* = 0.83).

Shifts in the intestinal microbiome are seen with SCD therapy. To date, with limited sample size, no consistent impact on the microbiome has been noted. In a study by Suskind et al., microbial diversity increased from baseline after two weeks for 4 out of 9 patients and had minimal/no change in 4 others. One patient experienced a large decrease observed after 2 weeks, mostly due to an expansion of *Faecalibacterium prausnitzii,* from 9.6% to 54%. Median diversity for the 9 patients showed only a moderate increase from 2.48 to 2.65. Changes in phylum abundance revealed a decrease in Proteobacteria in all patients with the exception of 2 patients, one of whose proteobacterial abundance was unusually high at 34% and one who had the lowest value at baseline of 0.3%. There was no net change in phyla over all patients as a group [54].

Recently, Shabat et al. conducted a randomized control trial for Ulcerative Colitis Exclusion Diet (UCED) with or without fecal microbiota transplantation (FMT) in adult patients who were refractory to medical therapy [58]. UCED by itself achieved higher clinical remission and mucosal healing than single-donor FMT with or without diet. The study was terminated early as per the safety monitoring board; however, 40% of patients on the UCED by itself went into clinical remission with endoscopic remission seen in 26% of patients at eight weeks. 

### 5.2. Low Fermentable Oligosaccharides, Disaccharides, Monosaccharides and Polyols (FODMAPs) Diet

FODMAPs consist of molecules that are poorly absorbed in the small intestine and are fermented by bacteria in the colon. These nondigestible components in foods potentially elicit symptoms in IBD patients that are similar to irritable bowel syndrome (IBS). Reported at a higher prevalence in patients with IBD, IBS is a functional gastrointestinal disorder characterized by a constellation of symptoms including abdominal pain, bloating, and diarrhea. Several studies in adult patients with CD and UC have demonstrated a low-FODMAP diet (LFD) reduced IBS-like symptoms and increased quality of life in patients [59,60,61]. 

Cox et al. completed a study in which IBD patients in remission with persistence of functional GI symptoms that met Rome III criteria for IBS, and who had experienced improvement following a LFD, were challenged with fructans [62]. The fructan challenge induced gastrointestinal symptoms in these patients, while galacto-oligosaccharides and sorbitol challenge did not. 

Cox et al. performed a single blind trial of quiescent IBD patients examining gut symptoms and health-related quality of life on LFD versus a control diet [63]. An increased percentage of patients reported the improvement of intestinal symptoms following the LFD (14/27, 52%) than the control diet (4/25, 16%, *p* = 0.007). Patients had a greater reduction in IBS severity scores following the LFD than the control diet, although this difference was not statistically significant. In addition, patients reported higher health-related quality of life scores when following the LFD (81.9 ± 1.2) than patients on the control diet (78.3 ± 1.2, *p* = 0.042).

Bodini et al. conducted a randomized 6-week trial of LFD or standard diet (SD) in 55 adult patients with IBD on biologic therapy and presence of functional GI symptoms that met Roma IV criteria. After the six-week dietary intervention, median HBi decreased in the LFD (4; IQR, 3–5 versus 3; IQR, 2–3; *p* = 0.024) but not in the SD (3; IQR, 3–3 versus 3; IQR, 2–4). 

While LFD shifts the intestinal microbiome, there is no clear impact on the diversity of the microbiome. In a meta-analysis of nine randomized control trials of a LFD in IBS, no consistent effect on microbiome metrics, including diversity, fecal SCFA concentrations, and fecal pH, was seen. With that being noted, there was an effect on the colonic microbiome specific to Bifidobacteria [64]. These findings are seen in IBS patients with quiescent IBD as well. LFD significantly lowered the abundance of Bifidobacterium adolescentis, Bifidobacterium longum, and Faecalibacterium prausnitzii more so than in patients on a control diet without any impact on microbial diversity and markers of inflammation [63].

These and other studies suggest FODMAP may be used in IBD patients who experience persistence of functional symptoms [65]. To date, there is no evidence that a low FODMAP diet lessens inflammation in active IBD. LFD should be considered only in quiescent IBD patients with active IBS. 

### 5.3. Mediterranean Diet

The Mediterranean diet (MD) is characterized by a higher consumption of vegetables, fruits, cereals, nuts, legumes, and unsaturated fat, with a moderate intake of fish and dairy, and reduced consumption of saturated fat, meat, and sweets. MD has been extensively studied outside of IBD and is associated with an array of benefits, including decreased cardiovascular disease and cancer, due to the postulated anti-inflammatory properties [66,67]. 

Chicco et al. conducted a six-month prospective interventional study in adults with IBD on MD with primary outcomes significant for decreased BMI and waist circumference, and reduced liver steatosis on liver ultrasound [68]. The anthropometric outcomes in this study were associated with spontaneous improvement of disease activity, measured by CDAI and Mayo score, decreased CRP and fecal calprotectin, and improved quality of life in CD and UC. Reduction of fecal calprotectin levels were also found in adult patients with UC after pouch surgery who followed the MD [69]. 

Strisciuglio et al. completed a cross sectional study in 53 pediatric patients with CD and 72 with UC from 2018 to 2019. The MD diet had a significant reduction in fecal calprotectin [70].

Lewis et al. completed a comparative study with 194 adults with CD over 12 weeks randomized to either the SCD or MD [57]. The results showed that SCD was not superior to MD. Both groups achieved symptomatic remission in over 40% of participants, with fecal calprotectin and CRP reduction being comparable. With regard to the intestinal microbiome, whole-genome sequencing was used to compare the impact of the two diets. The richness and Shannon’s diversity were comparable between the groups and remained stable throughout the study. Beta diversity changed slightly over the course of the study. This was not related to the diet or symptomatic remission but was weakly associated with the fecal calprotectin concentration. The authors suggested that given the greater ease of MD and other associated health benefits, MD may be preferred for patients with mild-to-moderate CD.

## 6. Conclusions and Future Direction

Dietary integration into the care of IBD patients is central to improving outcomes. Understanding and better defining dietary interventions for the induction of remission and maintenance in IBD is paramount in refining personalized medicine. Current research supports therapeutic diets in IBD. Despite this, dietary therapies are not routinely integrated into patient care. 

As with medical intervention, the utilization of dietary therapies requires thoughtful discussion. Patients must follow a dietician and have clearly stated goals and expectations. Whether diet is being used as a primary intervention, such as EEN, PEN with CDED or SCD, or a secondary intervention, such as MD, development of a patient specific plan is essential. 

While research in diet and IBD has expanded greatly in the last decade, much is still unknown. Future studies need to expand on the clinical application of diet in IBD as well as give better understanding of the mechanism by which diet works. Pairing immunology and microbiology by assessing specific immunologic cell and cytokine and chemokine profiles, growth and nutrition mediators, metagenomics, and nutrigenomics would give further insight into the mechanisms by which diet impacts IBD. With further research and integration of diet into clinical practice, dietary interventions have the potential to change the IBD paradigm.

## Figures and Tables

**Table 1:** Dietary interventions for Crohn’s Disease and Ulcerative Colitis.

Diet	Crohn’s Disease	Ulcerative Colitis
**EEN**	**+**	
**PEN/CDED**	**+**	
**SCD/MSCD**	**+**	**+**
**FODMAP**	**+**	**+**
**MD**	**+**	**+**

**Table 2:** Descriptor of dietary interventions for IBD.

Diet	Include	Avoid
Exclusive Enteral Nutrition	Nutritional complete formula with variable nutrient composition	All other nutritive sources
Partial Enteral Nutrition with Crohn’s Disease Exclusion Diet	Induction: First six weeks 50% of calories from formula, followed by 25% in weeks 7–12. Rest of calories CDED allowed foods including fruits, vegetables, meat, grains, oats, rice Maintenance: once in remission, continue allowed foods	Seafood other than fish, dairy, processed foods, artificial sweeteners, emulsifiers, cocoa, coffee, and alcohol
Specific Carbohydrate Diet	Whole food diet with emphasis on fruits, most vegetables, fresh legumes, meat, seafood, hard cheeses, yogurt fermented greater than 24 h	Grains, starchy vegetables, most dairy, processed foods, artificial sweeteners, emulsifiers, cocoa, sugars outside of honey
Low FODMAP diet	Certain fruits and vegetables, low lactose dairy, gluten-free grains	Certain fruits and vegetables high in fructose, fructans, and polyols, grains, most legumes high in galacto-oligosaccharides, dairy
Mediterranean diet	Whole food diet with emphasis on fruits, vegetables, whole grains, legumes, seafood, nuts, olive oil	High red meat intake, sweets, sugar, processed meat, dairy
